# Murine Gbp1 and Gbp2 are ubiquitinated independent of *Toxoplasma gondii* infection

**DOI:** 10.1186/s13104-018-3267-z

**Published:** 2018-03-06

**Authors:** Vesela Encheva, Clémence Foltz, Ambrosius P. Snijders, Eva-Maria Frickel

**Affiliations:** 1 Mass Spectrometry and Proteomics Platform, The Francis Crick Institute, 1 Midland Road, London, NW1 1AT UK; 2 Host-Toxoplasma Interaction Laboratory, The Francis Crick Institute, 1 Midland Road, London, NW1 1AT UK

**Keywords:** *Toxoplasma gondii*, Guanylate binding proteins, Ubiquitinated substrates, Ubiquitin

## Abstract

**Objective:**

The intracellular parasite *Toxoplasma gondii* can invade any nucleated cell residing inside a parasitophorous vacuole (PV). Upon infection, the cytokine interferon gamma (IFNγ) is produced and elicits host defence mechanisms able to recognise the PV and destroy the parasite. Hereby, Guanylate binding proteins, ubiquitin and the E3 ubiquitin ligases Tripartite Motif Containing 21 (TRIM21) and TNF receptor associated factor 6 are targeted to the murine PV leading to its destruction. This study is the side product of research aiming to identify ubiquitinated substrates in a TRIM21-dependent fashion in murine cells infected with *Toxoplasma*.

**Results:**

We infected IFNγ-stimulated murine embryonic fibroblasts (MEFs) from either C57BL/6×129 wild-type (WT) mice or C57BL/6 TRIM21^−/−^ mice with *Toxoplasma*. Using mass spectrometry, we analysed proteins in both cell backgrounds presenting with the di-glycine remnant of ubiquitination. In addition, we compared peptide levels between WT and TRIM21^−/−^ cells. In line with earlier reports, Gbp1 was expressed to higher levels in the C57BL/6×129 WT MEFs compared to the C57BL/6-only background TRIM21^−/−^ MEFs. Protein expression differences in these different murine backgrounds thus precluded identification of TRIM21-dependent ubiquitinated substrates. Nevertheless, we identified and confirmed Gbp1 and Gbp2 as being ubiquitinated in a *Toxoplasma*-infection independent manner.

**Electronic supplementary material:**

The online version of this article (10.1186/s13104-018-3267-z) contains supplementary material, which is available to authorized users.

## Introduction

*Toxoplasma gondii* is an apicomplexan parasite capable of infecting all warm-blooded animals. Felines are the definitive host of the parasite enabling sexual recombination, while mice present a natural intermediate host. Global human infection rates are 30% [1]. Immunocompetent people control the infection, however, *Toxoplasma* can cause congenital abnormalities, ocular disease and health problems in the immunocompromised [2].

*Toxoplasma* invades any nucleated cell residing in a PV [3]. The cytokine IFNγ is central in controlling *Toxoplasma* by highly upregulating host defence proteins, amongst them the Immunity Regulated GTPases (IRGs) and Gbps. Gbps are a class of 11 active large GTPases. Gbps disrupt the *Toxoplasma* PV membrane and expose the parasite to elimination by the host [4, 5]. Different *Toxoplasma* strains (e.g. type I, II and II are the North American and European strains) vary in their genomes and thus elicit divergent host responses. Gbps for example recognize type II and III PVs, but not type I PVs, a feature driven by *Toxoplasma* virulence factors [6].

IFNγ-dependent targeting of type II and III *Toxoplasma* PVs by ubiquitin in both mouse [7, 8] and human cells [9, 10] leads to *Toxoplasma* control. E3 ubiquitin ligases catalyse the transfer of a ubiquitin molecule from the E2 ubiquitin-conjugating enzyme onto the protein substrate [11]. A proportion of the ubiquitin around *Toxoplasma* PVs in murine cells is deposited there by the E3s TRIM21 and TRAF6 [7, 8]. TRIM21 is critically important for survival of *Toxoplasma* infection in vivo and is linked to the production of cytokines and parasite clearance, as well as the levels of K63-linked ubiquitin around the PV [8]. The ubiquitin/TRIM21/TRAF6 system targets the same vacuoles recognized by the Gbps, a process that is seeded by IRGs [7, 12].

Ubiquitinated protein substrates in murine or human cells infected with *Toxoplasma gondii* are unknown. The consequence of ubiquitination of host defence proteins could be manifold—these proteins could participate in immune-mediated signaling or be targets of pathogen defence strategies. Knowledge of these ubiquitinated substrates could lead to novel anti-*Toxoplasma* therapeutic strategies in humans. Since we currently do not know of any E3 ubiquitin ligase responsible for targeting the *Toxoplasma* PV in human cells, research along these lines is limited to murine systems.

Here, we aimed to find ubiquitinated substrates in a TRIM21-dependent fashion in murine cells infected with *Toxoplasma.* The goal was to identify *Toxoplasma* PV-resident ubiquitinated proteins that played important parts in pathogen defence. This study is the side product of our identification of TRIM21 as an essential player in murine host-mediated resistance to *Toxoplasma* in vivo [8]. Protein expression difference in the divergent murine backgrounds of the wild-type versus TRIM21^−/−^ cells precluded identification of TRIM21-specific substrates. However, we present the single observation of analysing and defining by mass spectrometry ubiquitinated substrates in IFNγ-treated *Toxoplasma*-infected WT and TRIM21^−/−^ MEFs. We identified and confirmed that Gbp1 and Gbp2 are ubiquitinated in WT MEFs in a *Toxoplasma*-infection independent fashion.

## Main text

### Methods

#### Cell culture, parasite culture and infection

WT and TRIM21^−/−^ MEFs were previously generated and cultivated in Dulbecco’s Modified Eagle Medium (DMEM) with 10% fetal bovine serum (FBS) at 37 °C and 5% CO_2_ [13]. Mice used for MEFs are on a project license approved by the Home Office, UK, under the Animals Scientific Procedures Act 1986 and the procedure was approved by the local ethical committee of the Francis Crick Institute Ltd. Although the *Gbp1* gene is present, following in vivo poly I:C stimulation, Gbp1 protein is not expressed in mice in a pure C57BL/6 background [14, 15]. Thus, WT MEFs were on a C57BL/6×129 background and derived from animal bred at the Francis Crick Insitute Ltd, while TRIM21^−/−^ MEFs were on a pure C57BL/6 background and purchased from the Jackson Laboratory. *Toxoplasma gondii* type I (RH) and type II (Pru) were maintained by serial passage on monolayers of human foreskin fibroblasts (HFFs) (ATCC, SCRC-1041) as described previously and were a gift from Jeroen Saeij (UC Davis, USA) [16]. A multiplicity of infection (MOI) of five was used after induction with 100 U/mL murine recombinant IFNγ (R&D Systems) for 16 h. For SILAC, cells were cultured in DMEM supplemented with light or heavy l-arginine and l-lysine. Unlabelled, hydrochloride forms of l-arginine and l-lysine (R0K0) were from Sigma-Aldrich (light isotopes). Hydrochloride forms of l-arginine [13C6, 15N4] and l-lysine [13C6, 15N2] (R10K8) were from CK Isotopes (CNLM-291-H-0.5 and CNLM-539-H-0.25). The infected monolayer of HFF was cultured in DMEM (light and heavy) supplemented with 1% dialysed FBS for six doublings. WT and TRIM21^−/−^ MEFs were cultured in both light and heavy media, supplemented with 10% dialysed FBS and passaged for five doublings.

#### Sample preparation for LC–MS/MS

DiGly peptides from Toxoplasma-infected MEFs were generated as previously described [17] with full details described in Additional file 1.

#### LC–MS/MS

Peptides were resuspended in 0.1% TFA and loaded on 50 cm Easy Spray PepMap column (75 μm inner diameter, 2 μm particle size, Thermo Fisher Scientific, ES803) equipped with an integrated electrospray emitter. Reverse phase chromatography was performed using the RSLC nano U3000 (Thermo Fisher Scientific) with a binary buffer system at a flow rate of 250 nL/min. Solvent A was 0.1% formic acid (Thermo Fisher, 28904, 5% DMSO (Sigma, 41640), and solvent B was 80% acetonitrile, 0.1% formic acid, 5% DMSO. The diGly enriched samples were run on a linear gradient of solvent B (2–40%) in 90 min, total run time of 120 min including column conditioning. The nanoLC was coupled to a Q Exactive mass spectrometer using an EasySpray nano source (Thermo Fisher Scientific).

The Q Exactive was operated in data-dependent mode acquiring HCD MS/MS scans (R = 17,500) after an MS1 scan (R = 70,000) on the 10 most abundant ions using MS1 target of 1 × 10^6^ ions, and MS2 target of 5 × 10^4^ ions. The maximum ion injection time utilised for MS2 scans was 120 ms, the HCD normalized collision energy was set at 28, the dynamic exclusion was set at 10 s, and the peptide match and isotope exclusion functions were enabled.

#### Data processing and analysis

The data were processed and analysed according to methods detailed in Additional file 1.

#### Immunoprecipitation and immunoblot

All steps were performed on ice. Cells were washed twice in PBS and lysed in ice cold lysis buffer (25 mM TrisHCl (Roche, 10708976001) pH 7.4, 5 mM MgCl_2_ (Sigma, M8266), 150 mM NaCl (Sigma, S7653), 0.5% NP40 (BDH Laboratory Supplies, 56009), cOmplete EDTA-free protease inhibitor cocktail (Sigma, 11836170001)). Total protein lysate was equalised between samples and 0.5 mg were immunoprecipitated with Ubiquitin 1 Tandem UBA (TUBE1) agarose (BostonBiochem, AM-125) according to the manufacturer’s recommendation. Immunoprecipitated proteins were eluted from the beads at 92 °C for 10 min in 1× NuPAGE protein loading buffer (Thermo Fisher, NP0008) supplemented with 1 mM DTT (Sigma, D5545). Samples were separated on a 4–12% gradient SDS-PAGE (Thermo Fisher, NP0322BOX). All of the immunoprecipitate was loaded and 5 μg of the original protein lysate per sample. Proteins were transferred onto nitrocellulose membrane (GE Health care Life Sciences, 10600001) using semi-dry immunoblotter (Biorad Trans-Blot SD Cell). Blots were blocked at RT for 1 h in 5% non-fat dried milk/PBS solution and probed with anti-Gbp1 (generated by us in [6]) and anti-Gbp2 antibodies (kind gift from Joern Coers, Duke; published in [18]) in 5% non-fat dried milk/PBS + 0.1% Tween-20 (Sigma, P1379). Blots were then incubated with HRP-conjugated anti-rabbit secondary antibody (Life Technologies, G21234) in 5% non-fat dried milk/PBS + 0.1% Tween-20 (Sigma, P1379) and developed with Pierce ECL Western Blotting Solution (Thermo Scientific, 32106).

### Results and discussion

The addition of the small protein ubiquitin to substrate proteins can alter the fate of these proteins and serve to send the protein to degradation or to induce cellular responses [19]. Ubiquitin as an anti-pathogen response has been widely studied, yet ubiquitinated protein substrates in murine or human cells infected with *Toxoplasma gondii* are unknown. Here, we aimed to identify ubiquitinated substrates in a TRIM21-dependent fashion in murine cells infected with *Toxoplasma*. We performed a stable isotope labelling with an amino acids (Silac) proteomics experiment in duplicate with Silac label reversal in *Toxoplasma*-infected murine cells. We compared IFNγ-stimulated wild-type (WT, C57BL/6×129) and TRIM21^−/−^ cells (C57BL/6) infected with type II *Toxoplasma*. Peptides originating from protein ubiquitination sites were affinity enriched using an antibody that is specific for lysine containing peptides with isolinked diglycine motifs as described elsewhere [20]. These peptides are generated after trypsin digestion of the cell lysate containing E3 ligase substrates. In comparing C57BL/6×129 WT MEFs with pure C57BL/6 TRIM21^−/−^ MEFs, we observed the predicted expression difference of Gbp1 protein between these divergent murine backgrounds. Cells from 129 mice had previously been classed as “responder” cells in terms of Gbp1 expression versus C57BL/6 belonging to the group of Gbp1 non-expressing and thus termed “non-responder” cells [14]. This distinction had been made on the basis of in vivo poly I:C stimulated mice [14]. Nevertheless, another report does find transcriptional upregulation of *Gbp1* after IFNγ stimulation of ANA-1 macrophages, derived from C57BL/6J mice [21]. The observed difference to our proteomics data (derived from IFNγ-stimulated MEFs) could be a cell-type dependent variation of Gbp1 expression (MEFs versus macrophages) or it is possible that the *Gbp1* transcripts that are observed in the ANA-1 macrophages do not translate into protein. Regardless, it is interesting to note that Gbp1 does exhibit expression differences in divergent inbred murine strains, while Gbp2 is more homogenously expressed between these murine backgrounds. The divergent protein expression of Gbp1 that was observed between the different murine background MEFs however also precluded our robust analysis if any of the ubiquitinated substrates were indeed TRIM21-dependent.

**Fig. 1:**
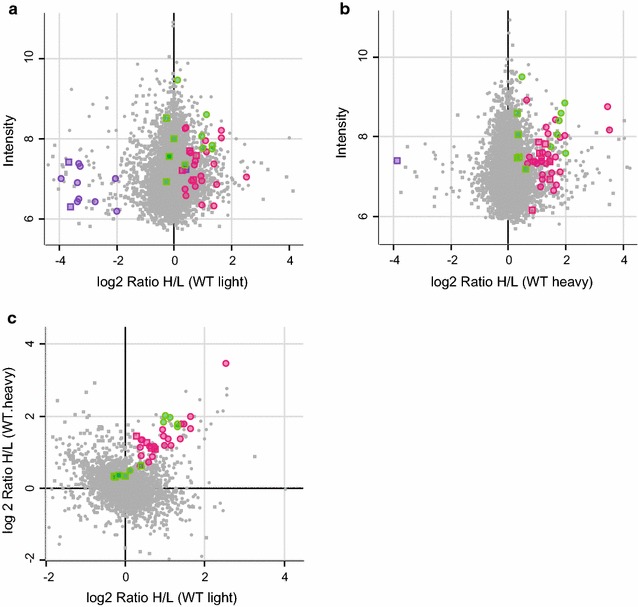
Mass spectrometry reveals Gbp1 and Gbp2 are ubiquitinated during *Toxoplasma* infection. **a**, **b** Ratio intensity plot and **c** correlation plot of peptide levels recovered by immunoprecipitation with a di-Gly antibody after trypsin digestion of IFNγ-stimulated C57BL/6×129 TRIM21 KO and wild-type C57BL/6×129 MEFs infected with *Toxoplasma.* Gbp1 and Gbp2 associated peptides both recovered as diGly-modified and- unmodified are marked. Gbp1 peptides are in purple and Gbp2 peptides are pink. Shared peptides are in green. Circles represent di-Gly modified peptides and squares are unmodified peptides

**Fig. 2:**
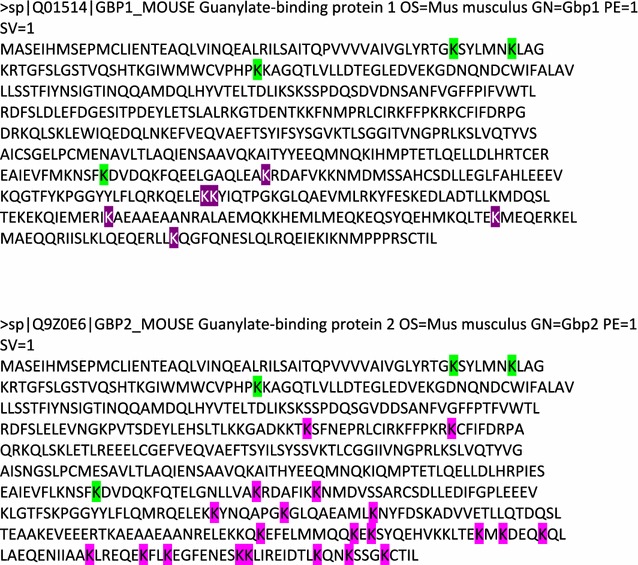
Identified ubiquitinated lysines of Gbp1 and Gbp2. FASTA sequence of Gbp1 and Gbp2 with the localisation of ubiquitinated lysines identified in this study marked. A total of 10 ubiquitinated lysines were found on Gbp1 including K51, K57, K87, K372, K389, K443, K444, K493, K532 and K560. Gbp2 had 25 modified lysines including K51, K57, K87, K215, K232, K372, K389, K396, K444, K451, K460, K509, K518, K520, K532, K534, K538, K551, K557, K560, K568, K569, K578, K581 and K585. Modified lysines found on peptides with identical sequences between Gbp1 and Gbp2 (shared peptides) are colored in green. Unique sites are colored in purple for Gbp1 and pink for Gbp2

Surprisingly, unmodified and di-Gly modified peptides derived from Gbp1 to Gbp2 demonstrated that both of these GTPases were themselves ubiquitinated in *Toxoplasma*-infected cells (Fig. 1, Additional file 2: Table S1). This is in contrast to Traver et al. who have shown that IRGs, but not Gbps, can be immunoprecipitated with a TUBE1 resin that specifically binds Lys63-linked ubiquitin [18]. For Gbp1 22 di-Gly modified peptides were identified which corresponded to 10 distinct ubiquitination sites (Fig. 2). For Gbp2 there were 36 di-Gly peptides and total of 25 ubiquitination sites (Fig. 2). The majority of the ubiquitination sites identified were localized in the C-terminal part of the proteins (Fig. 2). Very few ubiquitination sites on Gbp1 and Gbp2 have been previously described in the literature. The only two known sites, which we also detected in this study, are K232 and K389 on Gbp2 (https://www.phosphosite.org).

Additionally, several other IFNγ-inducible GTPases were found to be ubiquitinated. These include four sites on Igtp1, 10 sited on Irgm1, 12 sites on Irgm2, and 15 on Gvin1 (Additional File 2: Table S1).

**Fig. 3:**
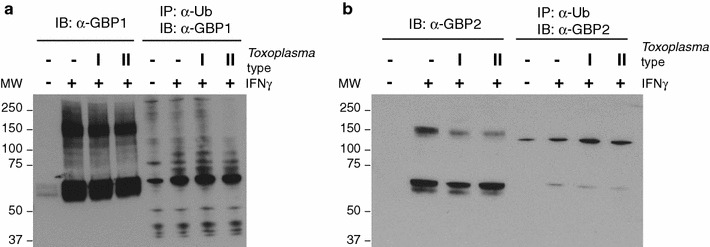
Confirmation of Gbp1 and Gbp2 ubiquitination by ubiquitin immunoprecipitation. **a**, **b** Immunoprecipitation using TUBE2 resin of ubiquitinated Gbp1 and Gbp2 proteins in IFNγ- or non-stimulated naïve or infected WT C57BL/6×129 MEFs. Lysate: 5 μg of the input. Data representative of three independent experiments. *MW* molecular weight (kDa)

In order to confirm that Gbp1 and Gbp2 are ubiquitinated, we analysed C57BL/6×129 MEFs stimulated with IFNγ and infected with *Toxoplasma* type I and II by immunoblot. We performed immunoprecipitation of ubiquitinated endogenous proteins followed by immunoblotting for Gbp1 and Gbp2. We detected both Gbps as being ubiquitinated (Fig. 3). Interestingly, Gbp1 and Gbp2 are both ubiquitinated regardless of cellular *Toxoplasma* infection status. Ubiquitination of Gbps has recently garnered attention in serving as a pathogen-mediated defence mechanism against these GTPases. A *Shigella flexneri*-encoded E3 ubiquitin ligase can target human GBP1 for ubiquitination and subsequent degradation, thus neutralising its original function of inhibiting actin-based bacterial mobility [22–24]. In our case, as the ubiquitination of Gbp1 and Gbp2 are *Toxoplasma*-infection independent, it remains to be investigated whether non-ubiquitinated versus ubiquitinated Gbp1 and Gbp2 would exhibit different localisation in the cell. It is conceivable that global Gbp1 and Gbp2 ubiquitination status might not change during infection, while this modification might target the defence proteins specifically to *Toxoplasma* PVs to excert their function.

## Limitations

As we are comparing MEFs from divergent murine backgrounds, we could not infer TRIM21-specific substrates from our experiments.

## Additional files

**Additional file 1.** Additional Methods.

**Additional file 2: Table S1.** List of all ubiquitinated protein sites identified in *Toxoplasma*-infected MEFs. Maxquant processed data taken from the modifiedpeptides.txt output table. Log2 normalised ratios for the indicated peptide sequences are displayed.

## Abbreviations

Gbp: guanylate binding protein
IFNg: interferon gamma
MEFs: murine embryonic fibroblasts
PV: parasitophorous vacuole
TRAF6: TNF receptor associated factor 6
TRIM21: Tripartite Motif Containing 21
WT: wild-type
FBS: fetal bovine serum
DMEM: Dulbecco’s Modified Eagle Medium
RH: *Toxoplasma* type I
Pru: *Toxoplasma* type II
MOI: multiplicity of infection
HFF: human foreskin fibroblasts
DTT: dithiothreitol
IAP: immunoaffinity purification
IRG: immunity related GTPases
RT: room temperature
TUBE1: Ubiquitin 1 Tandem UBA
