# Supplementary material for: Murine Gbp1 and Gbp2 are ubiquitinated independent of Toxoplasma gondii infection
Source: BMC Res Notes. 2018 Mar 6;11:166. doi: 10.1186/s13104-018-3267-z (PMC5840767; doi:10.1186/s13104-018-3267-z)
Supplement: Supplementary file 1 — Additional file 1. Additional Methods. [file 13104_2018_3267_MOESM1_ESM.docx]

**Additional Methods**

*Quantitative diGly Proteomics*

Cells were lysed in 9M urea (Fisher Chemicals, U/0500/53), 20mM HEPES (Sigma, H3375), pH=7.8, supplemented with 100units/ml of benzonase (Sigma, E1014) and sonicated to reduce viscosity (3mm probe, 50% amplitude, 3 x 15s bursts, on ice). 15 - 20mg of protein per sample was used as estimated by Bradford assay. Lysates were reduced with 10mM dithiothreitol (DTT) (Sigma, D5545) for 30min at room temperature (RT), followed by alkylation with 20mM chloroacetamide (Sigma, C0267) for 30min at RT in the dark. Lysates were digested initially with LysC (Promega, V1671) for 2h at 37⁰C. The lysates were then diluted with 100mM ammonium bicarbonate (Sigma, 70221, 5% acetonitrile to a final urea concentration of less than 2M. The samples were digested 1:100 enzyme to protein ratio (w/w) with trypsin (Promega, V5280) overnight at 37⁰C. The next day, two additional aliquots of trypsin were added and incubated at 37⁰C four hours each. After the digestion, the samples were acidified with TFA (Thermo Fisher Scientific, 28904) to final concentration of 1% (v/v). All insoluble material was removed by centrifugation and the supernatant was desalted with Sep-Pak C_18_ cartridges (Waters, WAT051910) and lyophilised for 2d.

Peptides containing the diGly remnant were enriched using K-ϵ-GG affinity resin (Cell Signaling Technology, 5526S) according to the manufacturer's instructions. Briefly, digests were reconstituted in 1.4ml of immunoaffinity purification (IAP) buffer as supplied by the manufacturer. One aliquot (∼40μl packed bead volume) was washed 4x with PBS and mixed with the peptide sample. Incubation of sample and beads was performed with gentle rotation at 4°C for 2h followed by a 30s 2000×g spin to pellet the beads. The antibody beads were washed twice with ice-cold IAP buffer followed by three washes with ice-cold water. DiGly peptides were eluted with the addition of 50μl of 0.15% TFA and allowed to stand at RT for 5min. After a 30s 2000×g spin, the supernatant was removed and retained for further analysis. A second 55μl aliquot of 0.15% TFA was added to the beads, spun at 30s 2000×g, and the supernatant was added to the first elution. The eluted peptides were lyophilised for 2d and used for SCX fractionation.

*SCX (strong cationic exchange) fractionation of diGly peptides*

Peptides eluted from the K-ϵ-GG affinity resin were dissolved in 35μl of 10mM ammonium formate (Sigma, 70221) pH=2.9, 25% acetonitrile (Thermo Fisher, A955-212), sonicated and insoluble material was removed by centrifugation at 21000xg. Peptide separation and fraction collection was performed using the micro pump on a RSLCnano U3000 (Thermo Fisher Scientific) at a flow rate of 50μl/min. The peptides were loaded on 15-cm Polysulfoethyl-Asp SCX column (1mm inner diameter, 5μm particle size, PolyLC, 164566). Solvent A was 10mM ammonium formate pH=2.9, 25% acetonitrile, and solvent B was 500mM ammonium formate pH=6.8, 25% acetonitrile. The samples were run on a linear gradient of 0-80% B in 45min, total run time was 75min including column conditioning. 30 fractions were collected every minute between 15-45min after injection (1 fraction= 50μl), vacuum dried and used for LC-MS/MS analysis.

*Data processing and analysis*

Raw data files were analysed with MaxQuant software (version 1.3.0.5) as described previously [1]. Parent ion and tandem mass spectra were searched against ToxoDB-12.0 *Toxoplasma gondii* ME49 and UniprotKB *Mus musculus* (August 2012) databases. A list of 247 common laboratory contaminants provided by MaxQuant was added to the database. The enzyme specificity was set to trypsin with maximum of three missed cleavages for the diGly datasets. The precursor mass tolerance was set to 20 ppm for the first search (used for mass re-calibration) and to 6 ppm for the main search. Product mass tolerance was set to 20 ppm. Carbamidomethylation of cysteines was specified as fixed modification, oxidized methionines and N-terminal protein acetylation were searched as variable modifications. Di-glycine-lysine was added to the list of variable modifications when samples enriched for diGly peptides were searched. The datasets were filtered on posterior error probability to achieve 1% false discovery rate on protein, peptide and site level.

**References**

1. Cox J, Matic I, Hilger M, Nagaraj N, Selbach M, Olsen JV, et al. A practical guide to the MaxQuant computational platform for SILAC-based quantitative proteomics. Nat Protoc. 2009;4:698–705.
